# Age-Dependent Dynamics of the Biliary Microbiome in Children with Choledochal Cysts: Functional Remodeling Underlying Taxonomic Conservation

**DOI:** 10.3390/pathogens15020147

**Published:** 2026-01-29

**Authors:** Xueqi Wang, Ran Duan, Anxiao Ming, Yifan Zhang, Tiezhu Liu, Xin Wang, Mei Diao

**Affiliations:** 1 Capital Institute of Pediatrics, Chinese Academy of Medical Sciences & Peking Union Medical College, Beijing 100020, China; iwangxueqi@gmail.com (X.W.);; 2 General Surgery Department, Capital Center for Children’s Health, Capital Institute of Pediatrics, Capital Medical University, Beijing 100020, China; 3 National Institute for Infectious Disease Control and Prevention, China CDC, Beijing 102206, China; 4 National Key Laboratory of Intelligent Tracking and Forecasting for Infectious Diseases, Beijing 102206, China; 5 National Institute for Viral Disease Control and Prevention, China CDC, Beijing 102206, China

**Keywords:** choledochal cyst, biliary microbiome, functional remodeling, age-dependent

## Abstract

Choledochal cyst (CC), a congenital biliary anomaly, is associated with recurrent infections, chronic inflammation, and an increased risk of malignancy. Although emerging evidence implicates the biliary microbiome in disease pathophysiology, its developmental dynamics in pediatric CC remain unclear. Using deep metagenomic sequencing and comprehensive functional annotation, this study characterized age-dependent changes in the biliary microbiome of 201 pediatric CC patients stratified into infancy (<1 year), early childhood (1–5 years), and later childhood (5–12 years). We found that while the taxonomic composition and alpha diversity of the microbiota remained conserved across age groups, profound functional remodeling occurred with host development. A core set of microbial species(*Bacteroidota*, *Actinomycetota*, *Bacillota*, and *Pseudomonadota*) and functional pathways was shared across all ages; however, early childhood (1–5 years) exhibited the greatest number of unique functional genes, metabolic pathways, and carbohydrate-active enzymes, identifying this period as a critical window for microbial metabolic adaptation. Age-specific patterns were also evident in clinically relevant traits: infants (<1 year) harbored the most unique antibiotic resistance and virulence factor genes, whereas the resistome and virulome became more streamlined in older children. These findings establish a paradigm of “taxonomic conservation coupled with functional remodeling” in the CC microbiome and highlight age as a key determinant of microbial community function. This study offers novel insights into the microbial dynamics underlying CC progression and suggests potential age-specific targets for future therapeutic strategies.

## 1. Introduction

Choledochal cyst (CC) is a congenital cystic dilatation of the biliary tree, representing one of the most common structural anomalies requiring surgical intervention in pediatric hepatobiliary practice [1,2]. While choledochal cyst (CC) is considered a rare anomaly in Western countries with an incidence of 1:100,000–1:150,000 live births, it presents a significantly higher health burden in East Asia, where the incidence is estimated at approximately 1:1000 [3,4]. This ethnic disparity is further evidenced by recent bibliometric analyses, which indicate that the majority of large-sample clinical studies on CC originate from Chinese medical centers [3]. Consequently, specialized tertiary centers in China often manage annual caseloads that exceed the cumulative lifetime experience of many Western institutions [5,6]. Beyond the mechanical implications of biliary obstruction, patients with CC are plagued by recurrent cholangitis, pancreatitis, and a profoundly elevated lifetime risk of biliary malignancy. The precise pathogenesis of CC [7,8], however, remains enigmatic. While genetic predispositions and anomalous pancreaticobiliary ductal union are recognized etiological factors, they do not fully explain the chronic inflammation, recurrent infections, and heterogeneous clinical progression observed in patients [9,10].

The biliary tract is now understood to host a complex microbial community, the biliary microbiome, which is increasingly implicated in the pathophysiology of various hepatobiliary diseases [7,11]. This raises a compelling, yet under-explored, hypothesis: the microbial inhabitants of the choledochal cyst may act as dynamic contributors to local inflammation, tissue remodeling, and disease complications. Preliminary studies employing 16S rRNA gene sequencing have hinted at the existence of a dysbiotic bacterial community within CC [12,13]. However, the field is significantly constrained by critical limitations: small sample sizes that hinder robust statistical conclusions, a predominant focus on taxonomic cataloging with limited functional insight, and a notable lack of age-stratified analyses [14,15,16]. The latter is particularly crucial, as pediatric CC spans a period of rapid host development—marked by immune system maturation, dietary transitions, and evolving environmental exposures—all of which are potent drivers of microbiome composition and function in other mucosal sites [17,18].

Thus, fundamental questions persist regarding whether the biliary microbiome in CC changes during child development [17,19] and, if so, whether such changes are confined to shifts in bacterial identity or extend to a deeper functional reprogramming of the microbial community in adaptation to the host’s age-specific physiology [20]. Addressing these questions necessitates moving beyond small-scale, descriptive studies to a large-scale, age-stratified, and functionally oriented investigation [21]. 

To bridge this knowledge gap, we conducted a deep metagenomic sequencing [8] study on a uniquely large cohort of 201 pediatric CC patients, stratified into infancy (under 1 year), early childhood (1–5 years), and later childhood (5–12 years). We hypothesized that while a core microbial structure might be conserved, the functional repertoire of the biliary microbiome undergoes significant, age-dependent remodeling. By integrating taxonomic profiling with comprehensive functional annotation, such as spanning core metabolism, carbohydrate utilization, antibiotic resistance, and virulence potential, this study aimed to establish a high-resolution functional map of the CC microbiome, assess age-related differences in community structure, and delineate the age-shared “core” versus age-unique “adaptive” features [22,23,24]. Our findings reveal a paradigm of “taxonomic conservation coupled with profound functional remodeling,” identifying early childhood (1–5 years) as a critical window for microbial metabolic adaptation and highlighting distinct, age-specific profiles of antibiotic resistance and virulence factors. This work provides novel mechanistic insights into CC pathophysiology and underscores the importance of age as a key variable in understanding and potentially managing the disease.

## 2. Materials and Methods 

### 2.1. Ethics Approval and Consent to Participate

This study was reviewed and approved by the Institutional Review Board of Capital Institute of Pediatrics, Chinese Academy of Medical Sciences & Peking Union Medical College (Approval No. SHERLL2024053, dated 22 September 2025). All procedures performed in studies involving human participants were in accordance with the ethical standards of the institutional and/or national research committee and with the 1964 Helsinki declaration and its later amendments or comparable ethical standards. Informed consent was obtained from the parents or legal guardians of all individual participants included in the study.

### 2.2. Study Cohort and Sample Collection

This study was approved by the Institutional Review Board of Capital Institute of Pediatrics, Chinese Academy of Medical Sciences & Peking Union Medical College (Approval No. SHERLL2024053). All procedures were performed in accordance with the ethical standards of the responsible committee and with the Helsinki Declaration. Written informed consent was obtained from the parents or legal guardians of all participating children. A total of 201 pediatric patients diagnosed with choledochal cyst, who underwent radical cyst excision surgery between April 2024 and May 2025, were retrospectively enrolled. During surgery, fresh tissue samples from the excised cyst wall were aseptically collected, immediately snap-frozen in liquid nitrogen, and stored at −80°C until DNA extraction. Patients were stratified into three non-overlapping age groups for analysis: under 1 year old (Under1, n = 86), from 1 to under 5 years old (Years1to5, n = 82, including 1-year-olds but excluding 5 year olds), and from 5 to 12 years old (Years5to12, n = 33). Relevant clinical metadata were retrieved from the hospital’s electronic medical record system. 

### 2.3. DNA Extraction, Library Construction, and Metagenomic Sequencing

Total genomic DNA was extracted from approximately 30 mg of each biliary tissue sample using the DNeasy Blood & Tissue Kit, (QIAGEN, Redwood City, CA, USA) according to the manufacturer’s protocol. DNA quantity and quality were assessed using a NanoDrop spectrophotometer (Thermo Fisher Scientific, Waltham, MA, USA) and Qubit fluorometer (Thermo Fisher Scientific, Waltham, MA, USA). For each sample, a metagenomic shotgun library with an average insert size of 350 bp was constructed using the NEBNext Ultra DNA Library Prep Kit (New England Biolabs, Ipswich, MA, USA). Paired-end sequencing (2 × 150 bp) was performed on the Illumina NovaSeq 6000 Xplus platform (Illumina, San Diego, CA, USA).

### 2.4. Bioinformatics Analysis

For raw data processing and quality control, raw sequencing reads were subjected to quality control using Fastp (v0.23.2, https://github.com/OpenGene/fastp, accessed on 27 January 2026) to remove adapters and low-quality reads. Human host-derived reads were identified and removed by mapping to the human reference genome (GRCh38) using Bowtie2 (v2.4.5, http://bowtie-bio.sourceforge.net/bowtie2/, accessed on 27 January 2026). For metagenomic assembly, gene prediction, and catalog construction. High-quality clean reads from each sample were de novo assembled individually using MEGAHIT (v1.2.9, https://github.com/voutcn/megahit, accessed on 27 January 2026) with default parameters. Contigs with a length ≥ 300 bp were retained for subsequent analysis. Open reading frames (ORFs) on these contigs were predicted using MetaGeneMark (v3.38, http://exon.gatech.edu/meta_gmhmmp.cgi, accessed on 27 January 2026). Predicted protein sequences with a length ≥ 100 amino acids were clustered at 95% identity and 90% coverage using CD-HIT (v4.8.1, http://www.bioinformatics.org/cd-hit/, accessed on 27 January 2026) to construct a non-redundant gene catalog. The abundance of each gene in each sample was calculated by mapping the clean reads back to the catalog using Bowtie2 and normalized to transcripts per million (TPM).

### 2.5. Taxonomic and Functional Annotation

For taxonomic assignment, the non-redundant gene catalog was aligned against the NCBI NR database using DIAMOND (v2.0.11) with a blastp e-value cutoff of 1 × 10^−5^. Taxonomic lineage was assigned using the lowest common ancestor algorithm in MEGAN (v6.21.5). Functional annotation was performed against multiple databases: Clusters of Orthologous Groups (COG), Kyoto Encyclopedia of Genes and Genomes (KEGG), Carbohydrate-Active enZYmes (CAZy), Comprehensive Antibiotic Resistance Database (CARD), Virulence Factors Database (VFDB), and MetaCyc. Annotations were obtained using EggNOG-mapper (v2.1.6) for COG and KEGG, dbCAN3 for CAZy, DeepARG for CARD, and DIAMOND searches against the respective databases for VFDB and MetaCyc, all with standard recommended parameters.

### 2.6. Statistical Analysis

Alpha diversity indices (observed species, Chao1, ACE, Shannon, Simpson) were calculated using the vegan package (v2.6-4) in R (v4.2.2) based on the gene abundance profile. Differences in alpha diversity indices among age groups were tested using the Kruskal–Wallis test. Venn diagrams were generated using the VennDiagram package to visualize shared and unique genes across age groups at both taxonomic (NR) and functional levels (COG, KEGG, etc.). All statistical tests were two-sided, and a *p*-value < 0.05 was considered significant.

## 3. Results

### 3.1. Study Cohort and Demographic Characteristics

**Figure 1:**
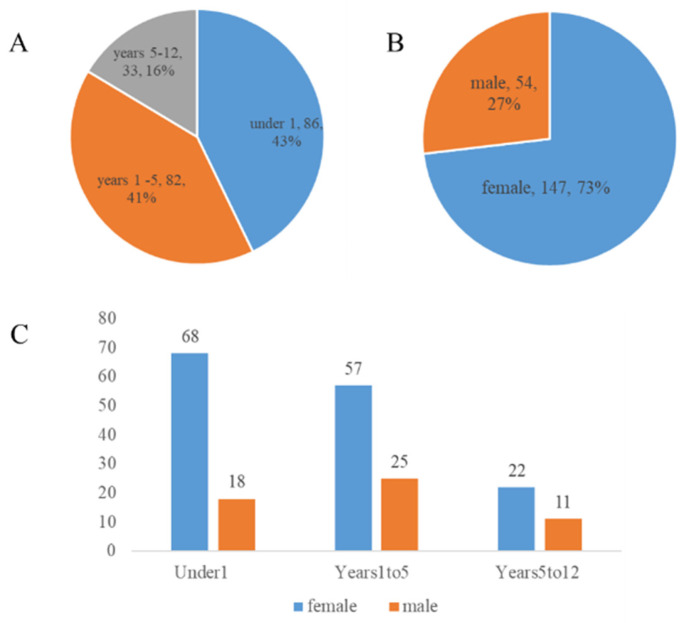
Demographic characteristics of the study cohort. (**A**). Age distribution of the 201 pediatric patients with choledochal cysts. (**B**). Sex distribution of the study cohort. (**C**). Distribution of study participants by age group and sex.

A total of 201 pediatric patients with choledochal cysts were included in this metagenomic study. The demographic composition of the cohort is detailed in Figure 1.

As shown in Figure 1A, the study population was stratified into three age groups for subsequent analysis. The under 1 year old group (Under1) constituted the largest proportion of the cohort (n = 86, 43%), followed closely by the 1 to 5 years old group (Years1to5, n = 82, 41%). The 5 to 12 years old group (Years5to12) accounted for the remaining 16% (n = 33). The overall sex distribution of the cohort is presented in Figure 1B, which reveals a notable female predominance. Female patients (n = 147) comprised 73% of the cohort, while male patients (n = 54) accounted for 27%, yielding an overall sex ratio (male: female) of approximately 1:2.7. Further stratification of the sex distribution within each age group is illustrated in Figure 1C. This detailed view confirms that the female predominance was a consistent trend across all three age groups, with the Under1 group having the highest absolute number of both female and male patients.

### 3.2. Quality Control and Characteristics of the Metagenomic Dataset

Rigorous quality control was performed on the metagenomic sequencing data obtained from 201 biliary samples. As shown in Figure S1A, the mean base quality score remained above Q30 for the vast majority of sequencing cycles, indicating a high per-base accuracy with an error rate of less than 0.1%. Figure S1B demonstrates a balanced and unbiased nucleotide composition, where the proportions of A, T, C, and G bases stabilized around 25% after the initial cycles, confirming the stability of the sequencing process.

Following assembly and gene prediction, we analyzed the length distribution of the predicted coding genes. The sequence length of the predicted genes exhibited a typical right-skewed distribution, with the majority of genes ranging between 400 and 1000 bp (Figure S1C). Finally, all predicted genes from all samples were clustered to construct a non-redundant gene catalog, which is fundamental for subsequent unified analysis. The length distribution of sequences within this catalog (Figure S1D) shows a similar profile, confirming the robustness of our gene set. This high-quality dataset provides a solid foundation for all downstream analyses of the biliary microbiome. Finally, the non-redundant gene catalog constructed from all 201 samples exhibited a broad length distribution (Figure S1E), encompassing a comprehensive set of microbial gene functions for subsequent comparative analyses across age groups. Together, these quality control metrics confirm the high reliability and suitability of the sequencing data for in-depth taxonomic and functional profiling.

### 3.3. Comprehensive Functional Profiling of the Biliary Microbiome

**Figure 2:**
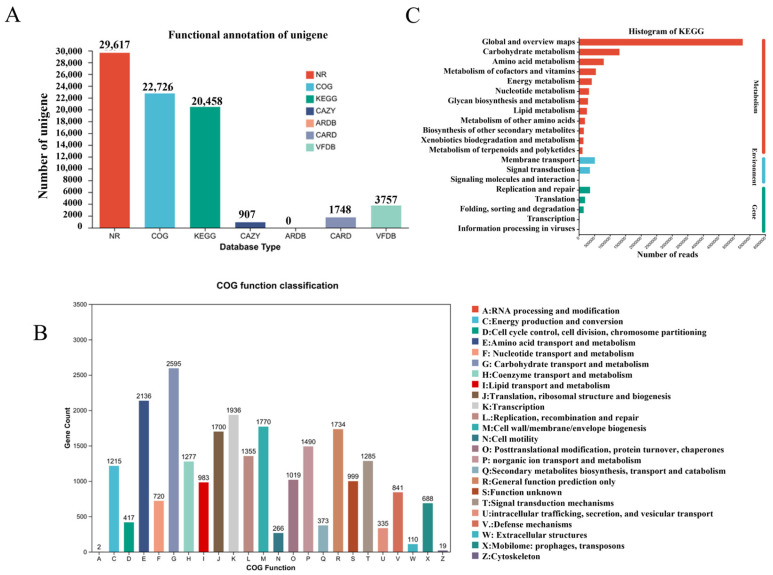
Overview of functional annotation and core metabolic profiling of the biliary micro-biome. (**A**). Success rates of functional annotation across major databases. (**B**). Functional classification of microbial genes based on the Clusters of Ortholo-gous Groups (COG) database. (**C**). Distribution of microbial functional potential across Kyoto Encyclopedia of Genes and Genomes (KEGG) pathways.

The functional potential of the biliary microbiome was extensively annotated and categorized, as summarized in Figure 2. Figure 2A details the overall success of the functional annotation process for the non-redundant gene catalog. The highest annotation rate was achieved against the NR database, with 29,617 genes annotated, establishing a robust foundation for taxonomic assignment. Substantial annotations were also obtained for general and specialized functional databases: 22,726 genes in COG, 20,458 genes in KEGG, and 907 genes in CAZy. Critically, genes implicated in clinically relevant traits were identified, including 1748 antibiotic resistance genes in the CARD database and 3757 virulence factor genes in the VFDB.

Functional categorization using the COG database revealed the predominant metabolic nature of the microbial community (Figure 2B). The most abundant categories were related to essential cellular processes, including amino acid transport and metabolism (Category E), transcription (K), replication and repair (L), and carbohydrate transport and metabolism (G). This profile indicates a community highly active in growth, maintenance, and energy acquisition.

At the pathway level, KEGG analysis further confirmed the dominance of core metabolic functions (Figure 2C). The most represented pathways belonged to “Metabolism,” with “Global and overview maps,” “Carbohydrate metabolism,” and “Amino acid metabolism” being the top three categories. Pathways involved in “Environmental Information Processing,” such as “Membrane transport” and “Signal transduction,” were also notably abundant, suggesting adaptive mechanisms for the biliary niche.

### 3.4. Specialized Metabolic Capabilities and Clinically Relevant Functional Traits

**Figure 3:**
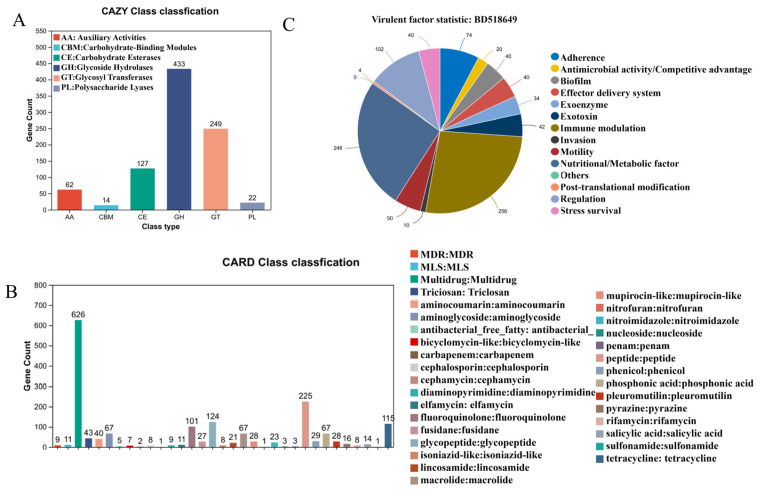
Specialized metabolic and clinically relevant functional features of the biliary micro-biome. (**A**). Repertoire of Carbohydrate-Active enZYme (CAZy) families identified in the biliary microbiome. (**B**). Composition and abundance of antibiotic resistance genes annotated from the Comprehensive Antibiotic Resistance Database (CARD). (**C**). Profile of virulence factor genes categorized using the Virulence Factors Database (VFDB).

Figure 3 presents a focused analysis on specialized metabolic functions and clinically relevant gene pools. A detailed analysis on carbohydrate-active enzymes (CAZy) highlighted a rich repertoire for carbohydrate metabolism (Figure 3A). Glycoside Hydrolases (GHs) and Glycosyl Transferases (GTs) were the two most abundant classes, underscoring a strong capacity for both the breakdown and synthesis of complex carbohydrates. A considerable number of genes encoding Carbohydrate-Binding Modules (CBMs) were also detected, which facilitate substrate recognition.

The annotation against the CARD database uncovered a diverse and concerning antibiotic resistome (Figure 3B). Genes conferring resistance to multiple drug classes (MDR) were highly abundant. More specifically, a high prevalence of genes encoding resistance to beta-lactam antibiotics—including penams, cephalosporins, and carbapenems—was identified, highlighting a significant challenge for clinical treatment.

Finally, the virulence factor profile was delineated using the VFDB (Figure 3C). Genes associated with “Biofilm” formation constituted the largest category, pointing to a key mechanism for persistence and antimicrobial tolerance. Substantial genetic potential was also found for “Exoenzyme” and “Exotoxin” production, “Immune modulation,” and “Adherence,” collectively outlining a multifaceted pathogenic strategy employed by the biliary microbiota.

### 3.5. Alpha Diversity Across Age Groups

**Figure 4:**
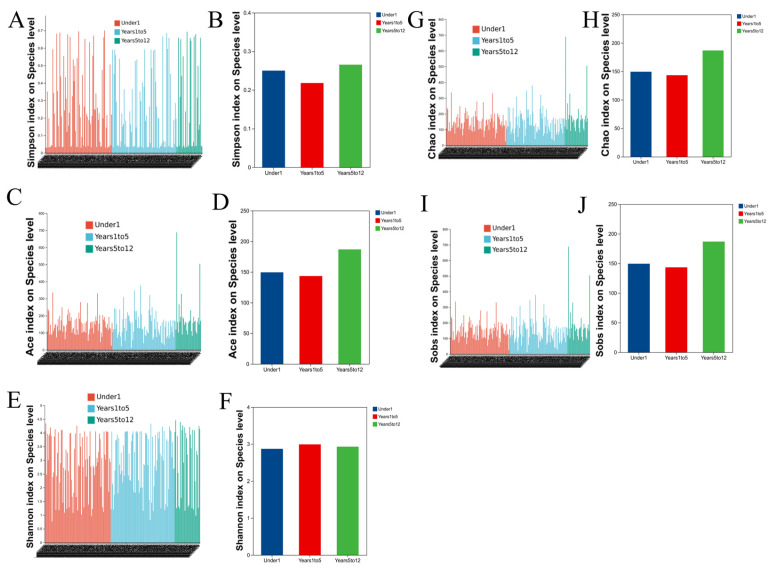
Alpha diversity of the biliary microbiome is comparable across pediatric age groups with choledochal cysts. Alpha diversity indices, including Simpson (**A**,**B**), ACE (**C**,**D**), Shannon (**E**,**F**), Chao (**G**,**H**), Sobs (**I**,**J**), show no significant differences among the three age groups.

Alpha diversity of the biliary microbiota, assessed across all major indices, did not exhibit statistically significant differences among the three age groups. Specifically, comparisons of species richness (Observed species (SOBS), Chao, and ACE indices) and diversity metrics that incorporate both richness and evenness (Shannon and Simpson indices) all yielded p-values greater than 0.05 in the Kruskal–Wallis tests (Figure 4). This indicates that the overall within-sample complexity, richness, and evenness of the microbial communities in the choledochal cyst were comparable in infants under 1 year, children aged 1–5 years, and those aged 5–12 years.

### 3.6. Age-Stratified Comparison of Taxonomic and Functional Gene Repertoires

**Figure 5:**
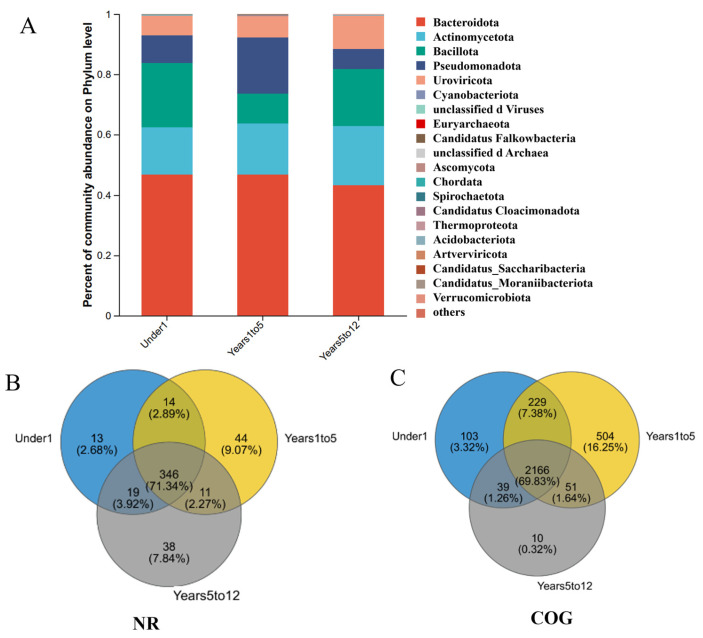
Taxonomic composition of the biliary microbiome at the phylum level across pediatric age groups. (**A**). Relative abundance of the top bacterial and archaeal phyla in the Under1 group (infants under 1 year, n = 86). (**B**). Relative abundance in the Years1to5 group (children aged 1–5 years, n = 82). (**C**). Relative abundance in the Years5to12 group (children aged 5–12 years, n = 33).

Microbiome profiling across age groups revealed distinct compositional patterns in choledochal cyst specimens (Figure 5A). At the phylum level, Bacteroidota, Actinomycetota, Bacillota, and Pseudomonadota constituted the predominant bacteria across all pediatric cohorts, collectively accounting for > 85% of the total microbial sequences.

To delineate the shared and age-specific components of the biliary microbiome, Venn diagram analyses were performed on the gene sets annotated from major taxonomic and functional databases. At the species level (Figure 5B, NR database), a substantial core microbiome of 346 species (71.34%) was common to all three age groups, while each group possessed unique species (Under1: 13; Years1to5: 38; Years5to12: 11). Analysis of general protein functions (COG database) revealed a highly conserved core functional repertoire of 2166 genes (69.83%) (Figure 5C). Strikingly, the Years1to5 group harbored the largest number of unique functional genes (229 genes, 7.38%), far exceeding those in the Under1 (103 genes) and Years5to12 (39 genes) groups. This pattern of high conservation coupled with age-specific features was consistent at the pathway level. 

KEGG pathway analysis showed an exceptionally high proportion of core pathways (331 pathways, 92.98%) shared across all ages (Figure S2A). Again, the Years1to5 group was distinguished by the greatest number of unique pathways (17 pathways, 4.78%). A similar trend was observed in the MetaCyc database of metabolic pathways, which identified 353 core pathways (62.15%) and highlighted the Years1to5 group as possessing the most unique metabolic pathways (115 pathways, 20.25%) (Figure S2B). Focusing on specialized metabolic capabilities, the CAZy database analysis identified a core set of 112 carbohydrate-active enzyme families (67.47%) (Figure S2C). Consistent with other functional analyses, the Years1to5 group contained the highest number of unique CAZy families (22 families, 13.25%). Clinically relevant gene pools also exhibited distinct patterns. The antibiotic resistome (CARD database) comprised a large shared core of 223 resistance genes (48.16%) (Figure S2D). The Under1 group featured the most unique resistance genes (60 genes, 12.96%). In contrast, the virulome (VFDB database) analysis showed a core of 286 virulence factor genes (57.66%) common to all groups (Figure S2E). Notably, the Under1 group possessed the largest unique set of virulence factors (58 genes, 11.69%).

## 4. Discussion

This study, through deep metagenomic sequencing of biliary tissue from 201 pediatric patients with choledochal cysts (CC), provides the first systematic characterization of age-dependent dynamics within the CC-associated biliary microbiome. Our central finding reveals a paradigm of “taxonomic conservation coupled with profound functional remodeling”. While the taxonomic composition and alpha diversity of the microbial community remained stable across age groups, its functional potential underwent significant and age-specific restructuring [25]. This pattern offers a novel microbial ecological perspective for understanding the chronic inflammatory and infectious processes in CC.

Firstly, the lack of significant differences in species composition and alpha diversity across the three age groups contrasts with the conventional view that host development markedly alters the microbiota at mucosal sites. This may suggest that the pathological structure of the CC provides a relatively isolated and stable physical and biochemical microenvironment, buffering the microbial community from the direct selective pressures associated with systemic host maturation, such as immune development and dietary shifts. However, in-depth functional analyses revealed a distinctly different dynamic landscape. The early childhood period (1–5 years) exhibited the greatest enrichment of unique functional genes, metabolic pathways, and carbohydrate-active enzymes (CAZys), indicating that this stage is a critical window for metabolic network reorganization and functional adaptation of the microbial community. This likely correlates with the transition from a milk-based diet to complex solid foods during this developmental period, prompting a functional “upgrade” of the microbiota to utilize new nutritional substrates.

Secondly, our analysis of the clinically relevant resistome and virulome revealed clear age stratification. Infants (<1 year) harbored the most unique antibiotic resistance genes (ARGs) and virulence factor genes (VFGs). This may reflect the selective pressure from early exposure to specific antibiotics in maternal or hospital environments, as well as the enhanced colonization and defense capabilities of initial colonizers establishing a niche in an immunologically immature host. In contrast, the resistome and virulome appeared to become more “streamlined” with age, with an increasing proportion of shared core genes. This may suggest that as the host immune system matures and the microbial community stabilizes, functional selection favors maintaining core ecological homeostasis over expressing redundant invasive traits. Notably, genes associated with biofilm formation were highly enriched across all age groups, strongly supporting biofilm formation as a core, conserved strategy for persistent colonization, resistance to bile flow, and antibiotic attack within the CC biliary environment.

Our findings elevate the role of microbes in CC from mere “infectious agents” to “dynamic modulators of disease progression.” Functional remodeling, particularly adaptive changes in metabolism and virulence, may directly participate in regulating the intensity of local inflammation, tissue repair processes, and potentially influence the long-term risk of complications such as biliary malignancy. This provides a potential microbiological explanation for the heterogeneous clinical progression observed in CC patients.

Furthermore, our analysis revealed significant differences in specific bacterial genera between age cohorts. However, we have deliberately refrained from overemphasizing these taxonomic shifts in our primary narrative for two principal reasons. First, the functional or pathogenic relevance of these specific taxa within the cyst microenvironment remains unverified, as our metagenomic data describe genetic potential rather than confirmed activity. Second, given the high dimensionality of species-level data, we prioritized the interpretation of broader, community-wide functional patterns which showed more systematic age-dependent remodeling and are less susceptible to statistical overinterpretation of sporadic taxonomic fluctuations.

This study has several limitations. The most significant is the lack of a true healthy pediatric biliary microbiome for comparison. Obtaining biliary samples from healthy children is nearly impossible due to profound ethical and practical constraints. Therefore, we cannot definitively conclude whether the microbial features observed here are unique to CC or partially reflect the developing biliary microbiome in normal physiology. Future studies could partially address this by comparing samples from patients undergoing surgery for other non-infectious, non-inflammatory biliary conditions or by employing advanced non-invasive sampling techniques for indirect inference [26,27]. Another notable limitation of this work is the lack of correlation between the metagenomic findings and key clinical parameters, such as serum bilirubin levels, cyst size, or histologic grades of inflammation. Future prospective studies integrating detailed clinical metadata with deep functional profiling are necessary to clarify how microbial functional adaptations are linked to disease presentation and outcome. Furthermore, while this study delineates age-stratified profiles of functional categories, metabolic pathways, CAZys, antibiotic resistance, and virulence factors, we acknowledge that a formal correlation analysis to directly link these specific functional modules was not performed. Such an analysis could elucidate whether, for instance, particular carbohydrate metabolism pathways co-occur with specific resistance mechanisms, potentially revealing ecological or genetic linkages within the cyst microenvironment, and we recognize its value in generating testable hypotheses regarding functional synergy. Critically, as a cross-sectional study, our findings reveal age-associated differences but cannot establish temporal sequence or causality. The observed pattern of “taxonomic conservation with functional remodeling” requires validation through longitudinal studies that track the same individuals over time, which would be essential to confirm the developmental dynamics and causal relationships implied by our data.

In conclusion, through large-scale, age-stratified, and functionally oriented analysis, this study maps the developmental dynamics of the biliary microbiome in pediatric CC. It establishes the core paradigm of taxonomic conservation with functional remodeling and identifies early childhood as a critical period for functional adaptation. These findings not only deepen the understanding of the microbial pathophysiology of CC but also suggest that future adjunctive microbiome-modulating strategies should consider patient age to achieve more precise management.

## Data Availability

The data supporting the findings of this study are openly available in Figshare at https://figshare.com/s/f6cc5febcff9a96a19d7 (accessed on 27 January 2026).

## Supplementary Materials

The following supporting information can be downloaded at: https://www.mdpi.com/article/10.3390/pathogens15020147/s1, Figure S1: Quality control and fundamental characteristics of the metagenomic dataset; Figure S2. Age-stratified Venn diagrams of functional and clinically relevant gene repertoires in the biliary microbiome
