# Supplementary material for: Age-Dependent Dynamics of the Biliary Microbiome in Children with Choledochal Cysts: Functional Remodeling Underlying Taxonomic Conservation
Source: Pathogens. 2026 Jan 29;15(2):147. doi: 10.3390/pathogens15020147 (PMC12943287; doi:10.3390/pathogens15020147)
Supplement: Supplementary file 1 [file pathogens-15-00147-s001.zip › pathogens-4079903-Supplementary.pdf]

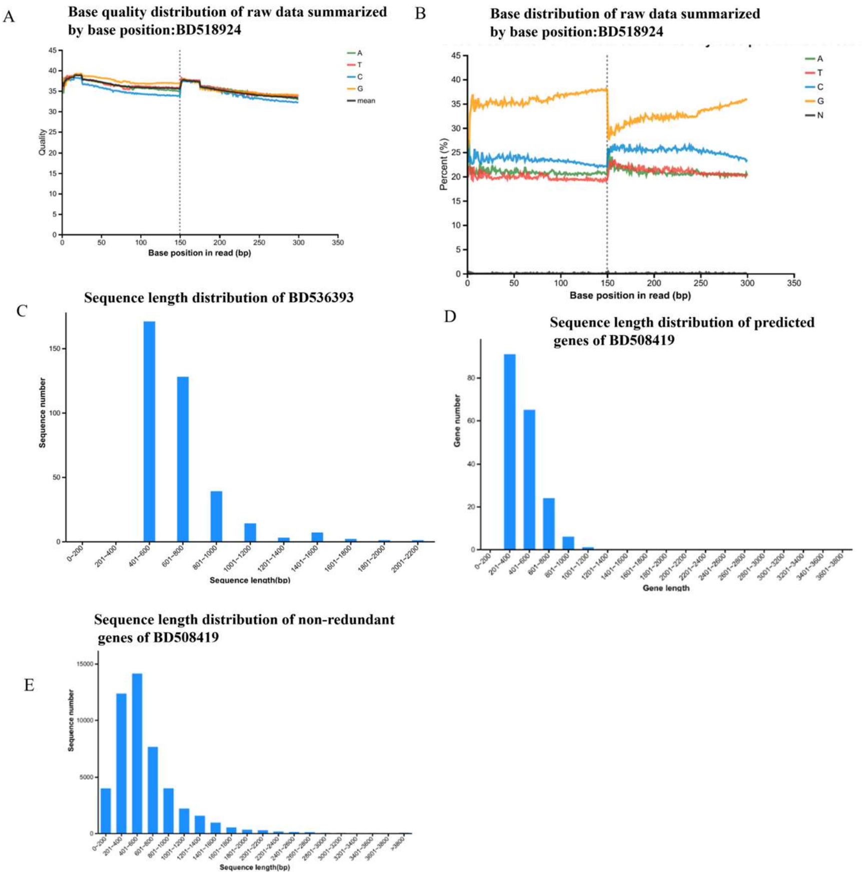

Figure S1. Quality control and fundamental characteristics of the metagenomic dataset. A. Per-base sequence quality across all sequencing reads. B. Nucleotide composition distribution along read positions. C. Length distribution of assembled contigs from a representative sample. D. Length distribution of predicted protein-coding genes from a representative sample. E. Length distribution of the non-redundant gene catalog constructed from all samples.

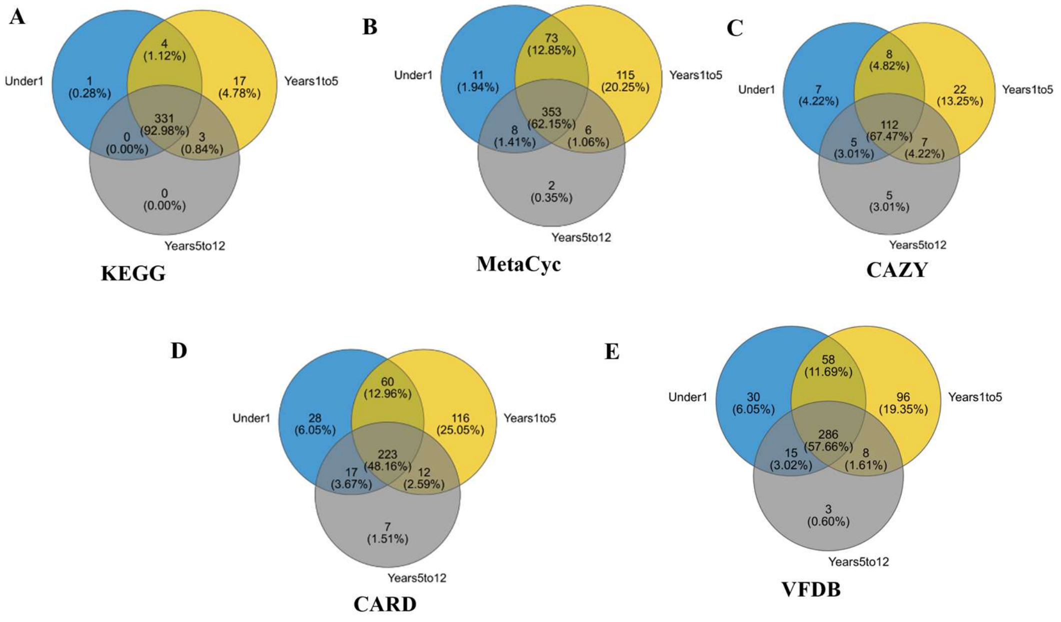

Figure S2. Age-stratified Venn diagrams of functional and clinically relevant gene repertoires in the biliary microbiome. A. Shared and unique KEGG pathways across age groups. **B**. Shared and unique metabolic pathways from the MetaCyc database. **C**. Shared and unique families of carbohydrate-active enzymes (CAZy) across age groups. D. Shared and unique antibiotic resistance genes (CARD database) across age groups. E. Shared and unique virulence factor genes (VFDB) across age groups.
